# Biological Characterization and Pluripotent Identification of Sheep Dermis-Derived Mesenchymal Stem/Progenitor Cells

**DOI:** 10.1155/2014/786234

**Published:** 2014-05-18

**Authors:** Peng Cui, Xiaohong He, Yabin Pu, Wenxiu Zhang, Ping Zhang, Changli Li, Weijun Guan, Xiangchen Li, Yuehui Ma

**Affiliations:** ^1^Institute of Beijing Animal Science and Veterinary, Chinese Academy of Agricultural Science, Beijing 100194, China; ^2^College of Pharmacy, Jiamusi University, Heilongjiang Province Key Laboratory of Biological Medicine Formulation, Jiamusi, Heilongjiang 154007, China

## Abstract

Dermis-derived mesenchymal stem/progenitor cells (DMS/PCs) were a multipotential stem cell population, which has potential applications in the tissue damage repair and skin transplant. Although a large number of studies deal with the human DMS/PCs self-renewal and regulation, however, the study of livestock-derived DMS/PCs has rarely been reported. Here, sheep DMS/PCs were isolated from one-month-old sheep embryos and studied at the cellular and molecular level. And then the DMS/PCs biological characteristics were analysed by RT-PCR and immunofluorescence. Experimental results showed that DMS/PCs could be expanded for 48 passages and the cells viability and hereditary character were steady. In addition, the DMS/PCs maker **β**-integrin, CD71, CD44, and CD73 were expressed positively through RT-PCR and immunofluorescence. Passage 3 DMS/PCs were successfully induced to differentiate into adipocytes, osteoblasts, chondrocytes, and neurocytes, respectively. The above results suggest that DMS/PCs not only have strong self-renewal capacity but also have the potential to differentiate into adipocytes, osteoblasts, chondrocytes, and neurocytes. The study provides theoretical basis and experimental evidence for potential clinical application.

## 1. Introduction

Mesenchymal stem cells (MSCs) are thought to be multipotent cells, which can replicate as undifferentiated cells and that have the potential to differentiate into lineages of mesenchymal tissues, including bone, cartilage, fat, tendon, muscle, and marrow [1]. Although MSCs can be greatly expanded in vitro and induced to differentiate into multiple mesenchymal cell types, however, differentiation to nonmesenchymal fate has been demonstrated. Woodbury performed a simple Induction system which was used to induce the stromal cells into neurocytes, expressing neuron-specific enolase, NeuN, neurofilament-M, and tau [2]. MSCs were first found in adult marrow. As MSCs derived from adult marrow possess pluripotency and proliferate extensively without obvious senescence or loss of differentiation potential, they may be an ideal cell source for therapy of inherited or degenerative diseases [3]. With more and more human diseases found, it is necessary to search various types of stem cells to serve human. In the recent years, MSCs were explored in the pancreas [4], umbilical cord blood [5], adipose [6], liver [7], and other tissues, which have a strong proliferative capacity and can differentiate into adipocytes [8–10], osteoblasts [11, 12], neurons [13–15], hepatocytes [16, 17], chondrocytes [18, 19], and so on.

Skin is a representative self-renewing tissue containing stem cells. As is accepted to us, there are many types of stem cells in the epidermis, dermis, and hair follicle to provide the ability of regeneration for skin; the stem cells in the skin tissue contain epidermal stem cell, dermal stem/progenitor cells, hair follicle stem cells, and the neurocytes derived from dermis tissue. Just due to the presence of these stem cells, our skin can maintain dynamic state for the long term. For the past few years, the stem cells isolated from dermis tissue have attracted great interest for their peculiar properties and potential applications in biological and medical fields. The forecast studies focus on the isolation and culture of stem cells from dermis, and the differentiation ability of the stem cells was found, which can differentiate into adipocytes, osteoblasts, chondrocytes, and neurocytes under the induction of small molecule compounds and the induced factors. Recently, dermal stem cells from caprine skin have shown promising characteristics for cartilage tissue engineering. These results show that dermal stem cells can be easily manipulated for cartilage tissue engineering strategies and may also be a useful cell source for another mesenchymal tissue [20].

Current research of stem cells focuses on humans, rabbits, mice, and other model animals. As the other animal model, the sheep possesses abundant dermal tissues. Moreover, the dermal stem cells can provide certain theoretical basis and experimental basis for the renewal of sheep skin and hair. Furthermore, the dermal stem cells can be applied in clinic for skin transplantation. In this study, we carried out a study on the isolation, culture, and differentiation potential of sheep DMS/PCs.

## 2. Materials and Methods

### 2.1. Experimental Animal

All the animal procedures were in accordance with The Institutional Animal Care and Use Committee of Chinese Academy of Agricultural Sciences. One-month-old sheep embryo was provided by the livestock and poultry Experimental Base Institute of Animal Sciences, Chinese Academy of Agricultural Sciences, Beijing. All the sheep embryos and all the experimental procedures involving sheep embryos for experimental research were followed with the protocols and guidelines for agricultural animal research codified by the Committee for Ethics of Beijing, China.

### 2.2. Experimental Reagents

The used reagents were as follows: low glucose DMEM (Gibco), fetal bovine serum (Hyclone), trypsin 1 : 250 (Amresco), rabbit anti-sheep *β*-integrin, CD71, CD44, CD73 polyclonal primary antibody (Abcam, America), rabbit to sheep *β*-III tubulin, NF, MAP-2 primary antibody, mouse to sheep nestin primary antibody (Santa Cruz, America), FITC-conjugated goat to rabbit secondary antibody IgG, FITC-conjugated goat to mouse secondary antibody IgG, TRITC-conjugated goat to rabbit secondary antibody IgG (Zhongshan Golden Bridge, China), dexamethasone (Sigma), indometacin (Sigma), *β*-glycerophosphate (Sigma), ascorbate (Sigma), tetracycline (Sigma), insulin (Sigma), 2-mercaptoethanol (Sigma), all-*trans*-retinoic acid (Sigma), alizarin red, Alcian blue (Boster, China), IBMX (Life Technologies, America), oil red O (Sigma), bFGF, EGF, TGF-*β*3 (Life Technologies, America), and dispase II (Gibco, America).

### 2.3. Isolation of DMS/PCs

About 25 cm square of dorsal skin tissues was isolated from one-month-old sheep embryos under sterile conditions. The samples were transferred into the benchtop and were washed about 6–8 times with phosphate buffered saline (PBS) including ampicillin (100 U/mL) and streptomycin (100 *μ*g/mL). Meanwhile, the fur of surface and adipose tissue of bottom from the dorsal skin tissues were cleaned in the PBS including antibiotics with sterile experimental apparatus, and the tissue samples were placed in 60 mm Petri dishes and cut into small pieces (1 cm^3^) using ophthalmic scissors. Then, the cleaned skin tissues were digested with 0.1% dispase II for 1.5–2 h at 37°C or overnight on the condition that the dermal layers were downward. After about 2 h or on the next day, the digested skin tissues were washed about 3–5 times with PBS; the dermal layer was isolated from the epidermal layers. The dermis was cut into approximately 1 mm^2^ pieces and then digested with 0.25% trypsin for about 20 min. Then, the enzymatic activity was terminated with equal DMEM containing 10% fetal bovine serum (FBS). The digested tissue was passed through a 200 *μ*m mesh filter and then centrifuged at 1200 r/min for 8 min at room temperature. The supernatant was discarded, and the pellet was resuspended with the optimized culture medium. After counting, cells were plated into flasks at 1 × 10^5^ cells/mL and cultured at 37°C, 5% CO_2_. After 24 h of culture, the cells were washed twice with PBS and nonadherent cells were removed. After about 2 days, DMS/PCs will grow fast at the confluence degree of 70–80%, and the cells were passaged with 0.25% trypsin and split into new flasks at the ratio 1 : 2 or 1 : 3. The cell type was inhomogenous among the first three passages, but, generally, after 3-4 passages, the cells were homogenous.

### 2.4. Optimization of Cell Culture Systems for DMS/PCs

DMS/PCs at the third passage were cultured in three types of culture systems at a density of 1 × 10^5^ cells/well onto 6-well plates: culture system I (L-Dulbecco's modified Eagle's medium [DMEM] supplemented with 10% FBS), culture system II (L-DMEM supplemented with 10% FBS, 2 mM L-glutamine, 1 mM sodium pyruvate, and 1% nonessential amino acid), and culture system III (L-DMEM supplemented with 10% FBS, 10 ng/mL epidermal growth factor [EGF], 10 ng/mL basic fibroblast growth factor [bFGF], 2 mM L-glutamine, 1 mM sodium pyruvate, and 1% nonessential amino acid). Cell morphology and cell vigour were observed every day and the generation time in each culture system was counted.

### 2.5. Estimation of Cell Viability

DMS/PCs viability was detected using the Trypan blue exclusion test [21] before and after cryopreservation as previously described. Cells were digested and seeded in 6-well plates and 1000 cells were stained and checked for cell viability rate.

### 2.6. Colony-Forming Cell Assay

Passage 3, passage 17, passage 31, and passage 45 cells were reseeded in 6-well plates at 100 cells/well and cultured for 7 days and then numbers of colony-forming units (CFU) were counted, to calculate colony-forming rate. This procedure was repeated six times for each passage number.

### 2.7. Growth Dynamics

By the traditional method, cells at final cell density of 1 × 10^4^/mL were seeded in triplicate in 24-well plates and cultured for seven days. Cell density data were monitored and recorded each day until the plateau phase. Three wells were counted each time, and the mean was deemed to a point of growth curve. The cell growth curve was then plotted. The population doubling time (PDT) was calculated based on the curve and the formula PDT = (*t* − *t*_0_)lg2/(lg*N*_*t*_ − lg*N*_0_),  *t*_0_: starting time of culture; *t*: termination time of culture; *N*_0_: initial cell number of culture; and *N*_*t*_: ultimate cell number of culture.

### 2.8. Identification of DMS/PCs Markers

#### 2.8.1. Reverse Transcription PCR (RT-PCR)

Passage 3, passage 23, and passage 43 cells were chosen as the low, middle, and high passages for RT-PCR; the cells were collected and the total RNA was extracted with Trizol reagent (Invitrogen). Total RNA was reverse transcribed to cDNA using PrimeScript 1st Strand cDNA Synthesis Kit (TARAKA, China). Specific gene primer pairs were designed and were listed in Table 1. PCR reaction was performed in a 20 *μ*L mixture containing 1.5 *μ*L template cDNA, 10 *μ*L Premix Taq (TARAKA, China), 1 *μ*L forward and reverse primers (TARAKA, China), and 7.5 *μ*L ddH_2_O. And the cycle conditions consisted of initial denaturation of 3 min at 94°C one cycle and then followed by 30 cycles of 30 s at 94°C for denaturation, 30 s at 55–65°C for annealing, and 30 s at 72°C for extending, finally, the last extension was performed for 10 min at 72°C for one cycle. PCR products were visualized by 2.5% agarose gel electrophoresis.

#### 2.8.2. Immunofluorescence

DMS/PCs were washed three times with PBS and fixed in 4% paraformaldehyde for 15 min and then washed three times in PBS (5 min each) again. Cells were permeabilized with 0.2% (v/v) Triton X-100 for 20 min and then washed further three times (5 min each) with PBS. The cells were blocked with 10% (v/v) normal goat serum (Santa Cruz Biotechnology, Santa Cruz, CA, USA) for 30 min and then incubated at room temperature for 1 h in 3% (w/v) bovine serum albumin (BSA) containing the following antibodies: rabbit anti-*β*-integrin (1 : 100; Abcam), rabbit anti-CD71 (1 : 100; Abcam), rabbit anti-CD44 (1 : 200; Abcam), rabbit anti-CD73 (1 : 200; Abcam), rabbit anti-*β*-III tubulin (1 : 200; Santa Cruz), rabbit anti-microtubule-associated protein-2 (MAP-2) (1 : 100; Santa Cruz), mouse antinestin (1 : 200; Santa Cruz), and rabbit antineurofilament (NF) (1 : 200; Santa Cruz) for 1 h at room temperature or overnight at 4°C. Next, the cells were washed three times (5 min each) with PBS and then incubated in PBS containing appropriate FITC-conjugated goat to mouse secondary antibody IgG or TRITC-conjugated goat to rabbit secondary antibody IgG for 1 h at 37°C. After incubation, cells were washed thrice with PBS (5 min each). The cells were observed using the Nikon TE-2000-E confocal microscope (Nikon, Yokohama, Kanagawa, Japan). Ten nonoverlapping visual fields were randomly selected. Images were acquired and used to calculate positive ratios. Cell nucleus was stained with DAPI (Sigma-Aldrich, St. Louis, MO, USA).

### 2.9. Karyotype Analysis

Passage 33 cells at the exponential growth phase were treated with 0.1 *μ*g/mL colcemid for 4 h at 37°C and were treated with a hypotonic KCl/HEPES/EDTA solution and were harvested and fixed. The chromosome numbers were counted from 100 spreads under an oil immersion objective upon Giemsa staining [22, 23]. The parameters of relative length, centromere index, and arm ratio index were calculated according to the current protocol.

### 2.10. Induced Differentiation of DMS/PCs

#### 2.10.1. Adipogenic Differentiation

Passage 3 cells which have higher differentiation potential were chosen for adipogenic differentiation. As the passage 3 DMS/PCs reached the confluence degree of 50–60%, DMS/PCs were divided into two groups: induced and control group. Cells of the induced group were incubated in adipogenic medium containing 10% FBS, 0.5 mM isobutyl-methylxanthine (IBMX; Life Technologies), 1 mM dexamethasone (Sigma), and 10 *μ*g/mL insulin (Sigma). Cells of the control group were incubated in complete medium without any induced factors. Medium was refreshed every three days. After 2 weeks of differentiation, oil red O staining was used for detection of accumulated oil droplets. And the specific makers of adipogenic cell including peroxisome proliferator-activated receptor *γ* (PPAR-*γ*) and lipoprotein lipase (LPL) were detected by RT-PCR analysis.

#### 2.10.2. Osteogenic Differentiation

Passage 3 DMS/PCs were reseeded in the 60 mm Petri plate at the density of 3 × 10^4^ cells/mL. At 50–60% confluence, the cells were divided into inducted and control groups. Cells of the induced group were incubated in osteogenic medium containing 10% FBS, 0.5 mM dexamethasone (Sigma), 10 mM *β*-glycerophosphate (Sigma), 60 *μ*M indometacin (Sigma), 2 mM L-glutamine, and 50 *μ*g/mL vitamin C. Cells in the control group were cultured in complete medium alone. The cell medium was refreshed every 3 days. After 2 weeks of differentiation, alizarin red staining was used to detect the presence of calcium node formation and the osteoblast-specific gene containing osteopontin (OPN), collagen type I (COLI), and alkaline phosphatase (ALP) was analyzed by RT-PCR.

#### 2.10.3. Cartilaginous Differentiation

Passage 3 DMS/PCs were reseeded in the six orifices at the density of 3 × 10^4^ cells/mL, when the confluence degree reached 70–80%, the cells were divided into inducted and control groups. Cells of the induced group were incubated in cartilaginous medium containing 10% FBS, 10 ng/mL transforming growth factor-beta 3 (TGF-*β*3), and 5 ng/mL bFGF (Life Technologies, America). Cells in the control group were cultured in complete medium. And the cell medium was changed every 2 days. About two weeks later, Alcian blue staining was used to detect the presence of cartilage condensations [24], and the osteoblast-specific gene containing collagen type II (COLII) was analyzed by RT-PCR.

#### 2.10.4. Neurogenic Differentiation

The DMS/PCs were seeded in the 6-well plates for neurogenic differentiation, and the cells were separated into two groups: inducted and control groups. When the confluence of cells reached 70%, the medium was changed. After the cells were washed three times, they were incubated in neurogenic cell inducted medium containing 10% FBS, 100 *μ*M 2-mercaptoethanol (Sigma), and 1 *μ*M all-*trans*-retinoic acid (Sigma) [25]. Cells in the control group were cultured in complete medium. And the cell medium was changed every 3 days. About 10 days later, neural-speciic markers including *β*-III tubulin, NF, nestin and MAP-2 in the inducted and control cells were detected through immunoluorescence.

## 3. Results

### 3.1. Morphology of DMS/PCs

**Figure 1:**
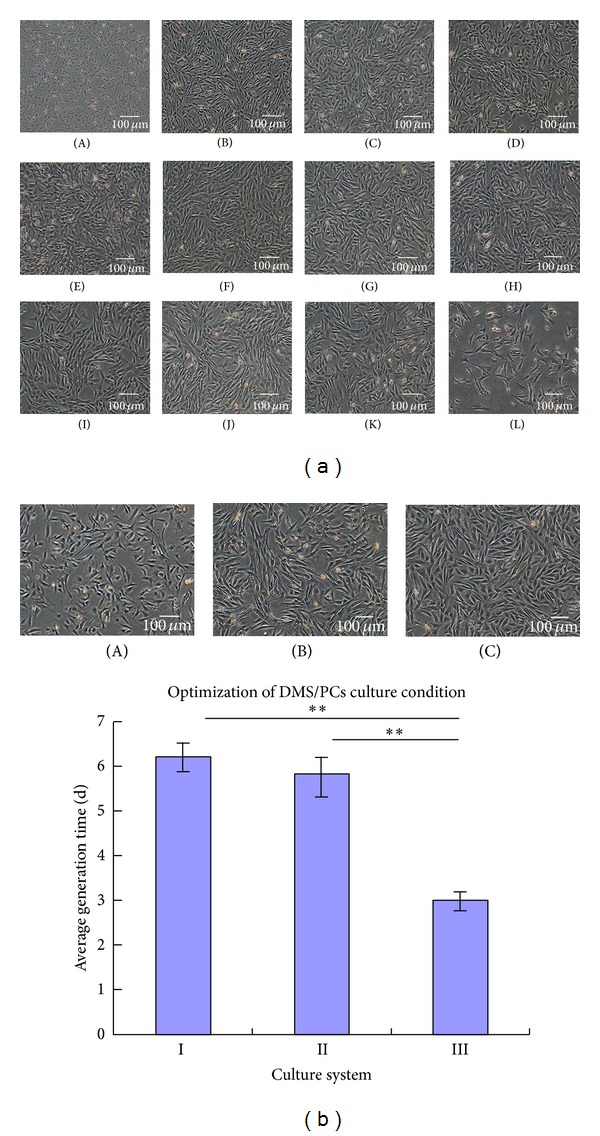
Morphology of DMS/PCs in vitro and optimization of DMS/PCs culture condition. (a) DMS/PCs of first three passages are inhomogenous. Afterwards, DMS/PCs were homogenous and the shape was spindle. (A) Primary cells after culture for 48 h. ((B)–(K)) Cell morphologies at passage 0, passage 5, passage 10, passage 15, passage 20, passage 25, passage 30, passage 35, and passage 40 before passage. (L) Cell morphologies at passage 48 (bar = 100 *μ*m). (b) ((A), (B), and (C)) Passage 3 DMS/PCs cultured in systems I, II, and III (bar = 100 *μ*m). The result showed that there was no significant difference between culture systems I and II on the cell vigour (*P* > 0.05), and for system III, there was significant difference compared to culture systems I and II (*P* < 0.01). The above results demonstrated that bFGF and EGF could maintain the self-renewing of DMS/PCs, and culture system III is optimal for proliferation of DMS/PCs.

The isolated DMS/PCs were seeded in the 60 mm Petri plate; after about 24 h, various shapes of cells began to adhere to the culture plates; the cell type is inhomogenous. About 48 h later, the cells began to elongate; a small number of polygonous cells could be also observed under the inverted microscope, and tiny minority of cells showed irregular shape. The cells proliferated quickly, displaying obvious karyokinesis, after about 5 days, the cells confluence degree could reach 80–90%; then, the cells were digested by 0.25% trypsin and 0.02% EDTA. The passage cells began to adhere after 2 h and completely adhered after 12 h. The passage cells grew rapidly, and the proportion of spindle-shaped cells also increased gradually. After three passages, the DMS/PCs were purified almost completely, and the shape was also homogenous. The cells were subcultured to passage 48 (Figure 1(a)), when the cells of passage 45 displayed representative senescent appearance as vacuole, tabular shape, and karyopyknosis in most cells, and the generation time increased with the increase of passage.

### 3.2. Optimization of In Vitro Culture Condition

Three different types of culture systems were selected to determine the optimal culture conditions for DMS/PCs; the cell morphology, cell vigour, and generation time were observed and detected in each culture system. The result showed that there was no significant difference between culture systems I and II on the cell vigour (*P* > 0.05), and the generation time was about 5 days for both systems. For system III, comparing with the above culture system, the cell morphology and cell vigour were good relatively, and the generation time was 2-3 days, which was significantly different compared to culture systems I and II (*P* < 0.01) (Figure 1(b)). The above results demonstrated that bFGF and EGF could maintain the self-renewing of DMS/PCs, and culture system III is optimal for proliferation of DMS/PCs.

### 3.3. Colony-Forming Cell Assay

**Figure 2:**
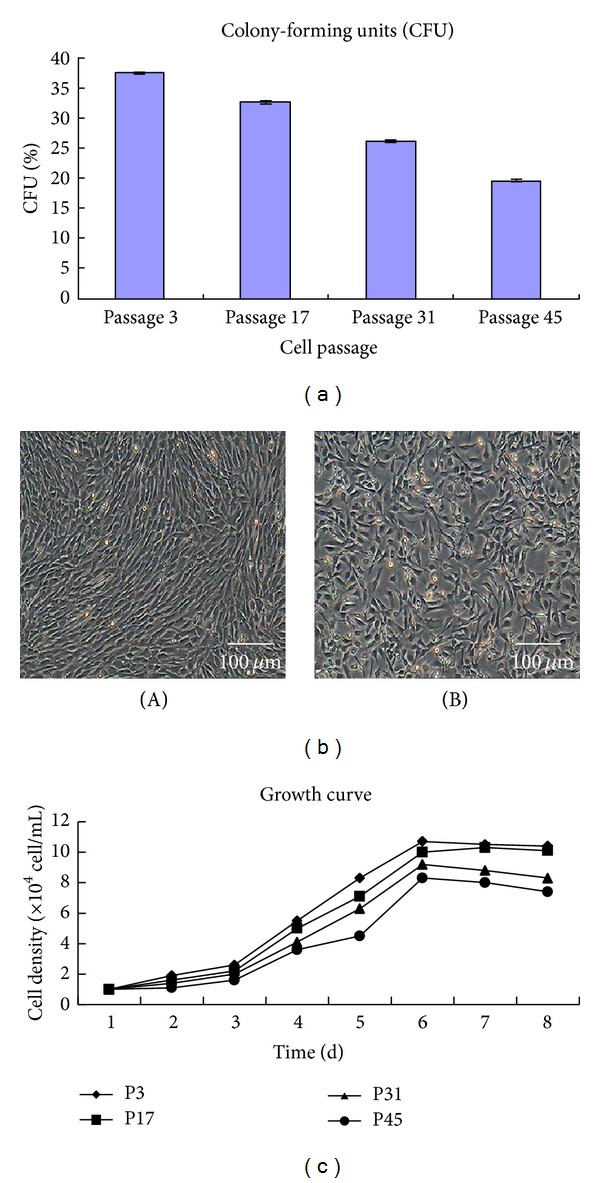
Detection of DMS/PCs vigour. (a) Colony-forming cell assay of DMS/PCs for passage 3, passage 17, passage 31, and passage 45, respectively, indicating the self-renewal ability of cultured DMS/PCs reduced gradually with the increase of passage number. (b) DMS/PCs before freezing and after recovery. (A) Before freezing; (B) after recovery (bar = 100 *μ*m). (c) Growth curve of different passages DMS/PCs. The growth curve of DMS/PCs appeared as typical “S” shape and the population doubling time (PDT) was 49.73 h, 50.36 h, 55.03 h, and 58.18 h for passage 3, passage 17, passage 31, and passage 45, respectively, on the base of the growth curve.

Colony formation was observed after culturing for 7 days by microscopy. The CFU were 37.3 ± 0.2%, 32.6 ± 0.3%, 26.6 ± 0.1%, and 19.6 ± 0.3% for passage 3, passage 17, passage 31, and passage 45, respectively (Figure 2(a)), indicating the ability of cultured DMS/PCs for self-renewal.

### 3.4. Cryogenic Preservation and Resuscitation

As the cell confluence degree reached about 90%, the cells were harvested by routine method. The harvested cell pellet was resuspended in a freezing medium containing 10% dimethylsulfoxide (DMSO), 50% FBS, and 40% L-DMEM to reach a final cell density of 4 × 10^6^ viable cells/mL, and finally the cells were transferred to liquid nitrogen for long-term storage. When recovered, the cells began to adhere after about 1 h and grew rapidly in very good health. The growth state and morphology were normal; there was no significant difference between cells before freezing and after recovery (Figure 2(b)). The cell motility rate was detected before freezing and after recovery. Cell viabilities were 98.5% ± 0.4% and 94.2% ± 0.2%, respectively. Therefore, the freezing storage conditions were appropriate and the DMS/PCs could be effectively preserved by the way.

### 3.5. Growth Kinetics

The proliferation rule of DMS/PCs at passage 3, passage 17, passage 31, and passage 45 was detected by cell hemocytometer, and the growth curve was drawn following the counting data (data not shown) (Figure 2(c)). Through analyzing the growth curve, we could see that there was a lag time or latency phase of about 24 h after seeding; then the cells proliferated rapidly and entered exponential phase. Along with the cell density increase, proliferation was restrained as a result of contact inhibition and the cells entered the plateau phase at approximately 6-7 days and began to degenerate. The PDT was 49.73 h, 50.36 h, 55.03 h, and 58.18 h for passage 3, passage 17, passage 31, and passage 45, respectively, on the base of the growth curve.

### 3.6. Identification of DMS/PCs

#### 3.6.1. Reverse Transcription PCR (RT-PCR) and Immunofluorescence

**Figure 3:**
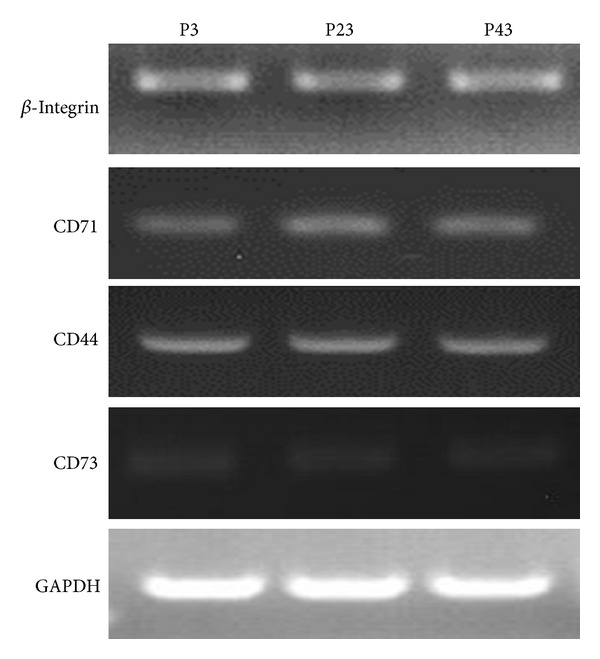
Detection of DMS/PCs markers by RT-PCR. The RT-PCR results show that *β*-integrin, CD71, CD44, and CD73 of P3, P23, and P43 DMS/PCs are positively expressed. GAPDH served as the internal control.

**Figure 4:**
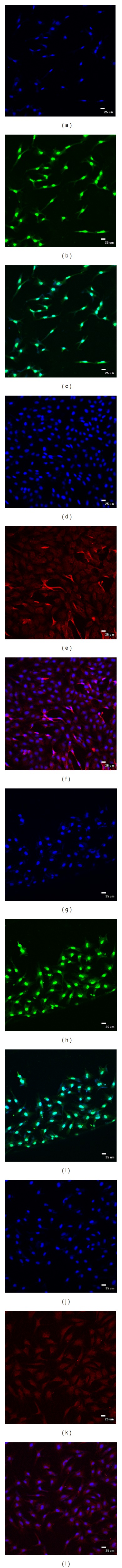
Detection of DMS/PCs markers by immunofluorescence staining. The immunofluorescence staining results show that *β*-integrin, CD71, CD44, and CD73 are positively expressed. ((a), (d), (g), and (j)) Blue staining represents DAPI counterstain of DMS/PCs nuclei. (b) *β*-Integrin+. (e) CD71+. (h) CD44+. (k) CD73+. ((c), (f), (i), and (l)) Merge (bar = 25 *μ*m).

The specific markers of DMS/PCs *β*-integrin, CD71, CD44, and CD73 were detected by RT-PCR and immunofluorescence. RT-PCR experimental results showed that the DMS/PCs were positive for *β*-integrin, CD71, CD44, and CD73, and GADPH was used as an internal control (Figure 3). The result of immunofluorescence showed that DMS/PCs specific markers *β*-integrin, CD71, CD44, and CD73 were positively expressed under the confocal microscopy (Figure 4).

#### 3.6.2. Karyotype Analysis

**Figure 5:**
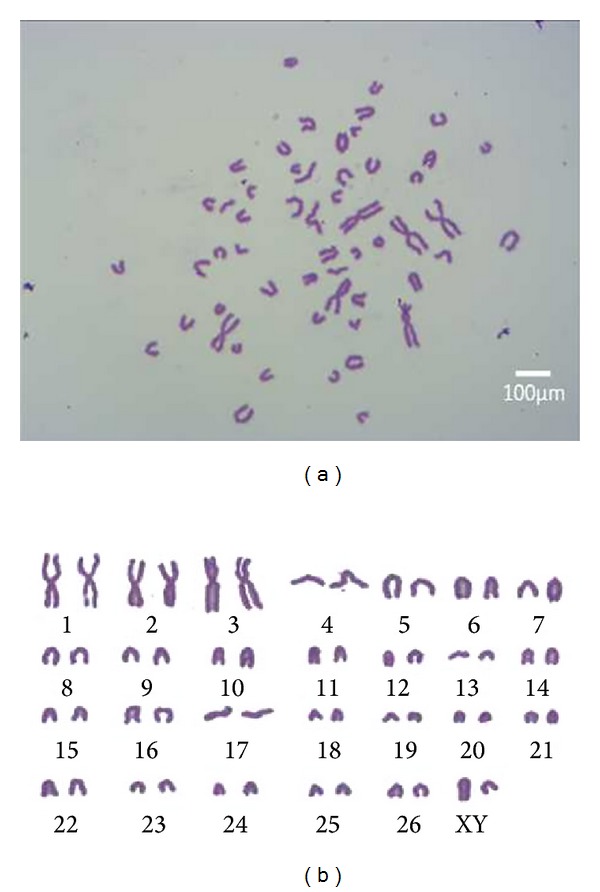
Karyotype analysis of DMS/PCs. Passage 33 cell chromosomes at metaphase (a) and karyotype (b) of DMS/PCs. The chromosome number of chicken was 2*n* = 54, including 26 pairs of euchromosomes and one pair of sex chromosomes, XY (bar = 100 *μ*m).

The chromosome number of sheep is 2*n* = 54, including 26 pairs of euchromosomes and one pair of sex chromosomes, XY (Figure 5). The X chromosome is the longest acrocentric chromosome and the Y chromosome is the shortest submetacentric chromosome. The chromosome numbers per spread were counted for 100 spreads of the passage 3, passage 23, and passage 45, and the ratio of cells with 2*n* = 54 was 93.5%, 92.6%, and 91.3%, respectively, implying that the cultured cells possessed genetic stability. The results also demonstrated that the chromosome number of individual cells tended to alter. And in vitro culture affected the hereditary property of cells slightly, but the evidence showed that the cell line was reproducibly diploid.

#### 3.6.3. Adipogenic Differentiation

**Figure 6:**
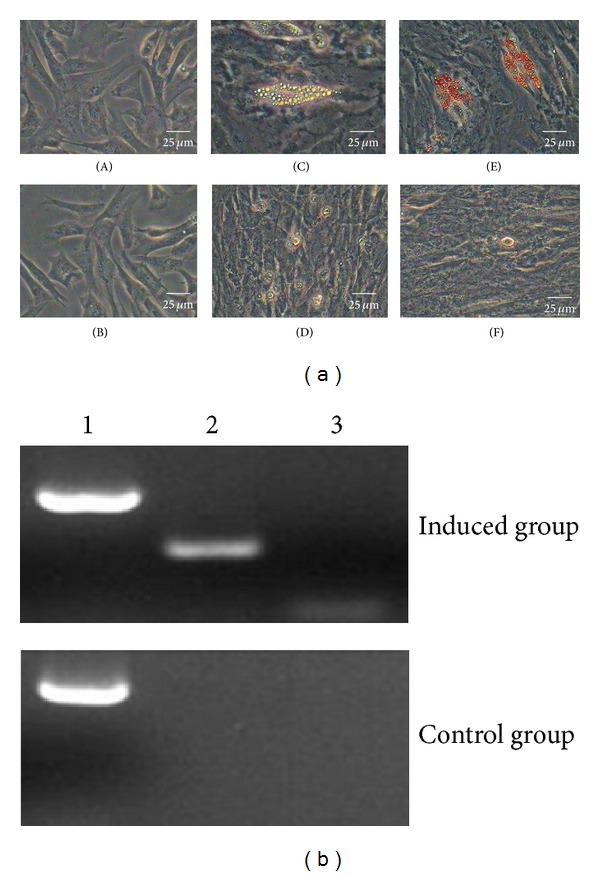
Adipogenic differentiation of DMS/PCs. (a) DMS/PCs morphology and staining detection of the induced group and the control group. ((B), (D), and (F)) The control group of adipogenic differentiation. After 1 d, 7 d, and 14 d, the cells cultured in complete medium had no difference in the morphology and phenotype and were also negative for oil red O staining. (A) DMS/PCs induced in the inducing culture medium after 1 d, and there is no difference in the morphology and phenotype. (C) The induced group of adipogenic differentiation. After induction for 7 days, the cell morphology began to change from a shuttle shape to an oblate shape, and many intracellular lipid droplets were present in the induced cells. (E) The cells of induced group were positive for oil red O staining (bar = 25 *μ*m). (b) RT-PCR detection of the adipogenic markers LPL and PPAR-*γ* expression in induced group and control group. Induced cells were positive for LPL and PPAR-*γ*, but the control cells were not. 1: GAPDH; 2: LPL; 3: PPAR-*γ*.

Passage 3 DMS/PCs at the confluence degree of 50–60% were used to differentiate into adipogenic cells in the induced medium, and the induced cells were detected by subsequent morphological and phenotypic analysis. After induction for 7 days, the cell morphology began to change from a shuttle shape to an oblate shape, and many intracellular lipid droplets were present in the induced cells. Along with the extension of induction time, the number of lipid droplets increased gradually and aggregated into the larger droplets (Figures 6(A)–6(C)). The differentiation result was tested by the way of oil red O staining; the induced cells were positive for the staining result (Figures 6(A)–6(E)). However, the cells which were cultured after 1 d, 7 d, and 14 d in the control group have no difference in the morphology and phenotype and were also negative for oil red O staining (Figures 6(A), 6(B), 6(D), and 6(F)).

In order to confirm that differentiation had occurred, the expression of adipogenic markers was examined in the induced and control group by RT-PCR. Induced cells were positive for the adipogenic markers PPAR-*γ* and LPL, but the control cells were not (Figure 6(b)). In addition, we could draw a conclusion that the upregulation of PPAR-*γ* and LPL gene expression played an important role in the adipogenic diferentiation. Above all, DMS/PCs can be induced to differentiate into adipogenic cells in vitro successfully.

#### 3.6.4. Osteogenic Differentiation

**Figure 7:**
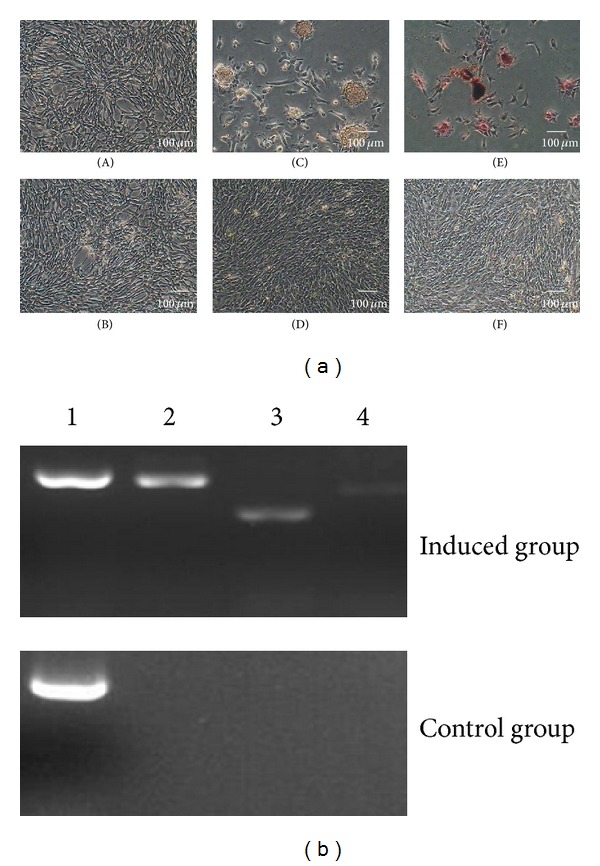
Osteogenic differentiation of DMS/PCs. (a) DMS/PCs morphology and staining detection of the induced group and the control group. ((B), (D), and (F)) The control group of osteogenic differentiation. After 1 d, 7 d, and 14 d, the cells cultured in complete medium had no difference in the morphology and phenotype and were also negative for alizarin red staining. (A) DMS/PCs induced in the inducing culture medium after 1 d, and there is no difference in the morphology and phenotype. (C) The induced group of osteogenic differentiation. After induction for 7 days, the cells became confluent and formed mineralized nodules. (E) The cells of induced group were positive for alizarin red staining (bar = 100 *μ*m). (b) RT-PCR detection of the osteogenic markers OPN, COLI, and ALP expression in induced group and control group. Induced cells were positive for OPN, COLI, and ALP, but the control cells were not. 1: GAPDH; 2: OPN; 3: COLI; 4: ALP.

The ability of sheep DMS/PCs to differentiate into osteogenic cells was also inspected, and the morphological and phenotypic analysis was carried on for the induced cells. The cells that were cultured after 1 d were put into the induced culture medium. The cell morphology was changed at seven days after induction; the cells became confluent and formed mineralized nodules which were bigger for further induction (Figures 7(A)–7(C)) and were positive for alizarin red staining (Figures 7(A)–7(E)), whereas the cells which were cultured after 1 d, 7 d, and 14 d in the control group did not show the above effects (Figures 7(A), 7(B), 7(D), and 7(F)).

The result of osteogenic differentiation of DMS/PCs was also analyzed by RT-PCR. Osteogenic-specific genes OPN, COLI, and ALP were expressed in the induced group, but not in the control group (Figure 7(b)).

#### 3.6.5. Cartilaginous Differentiation

**Figure 8:**
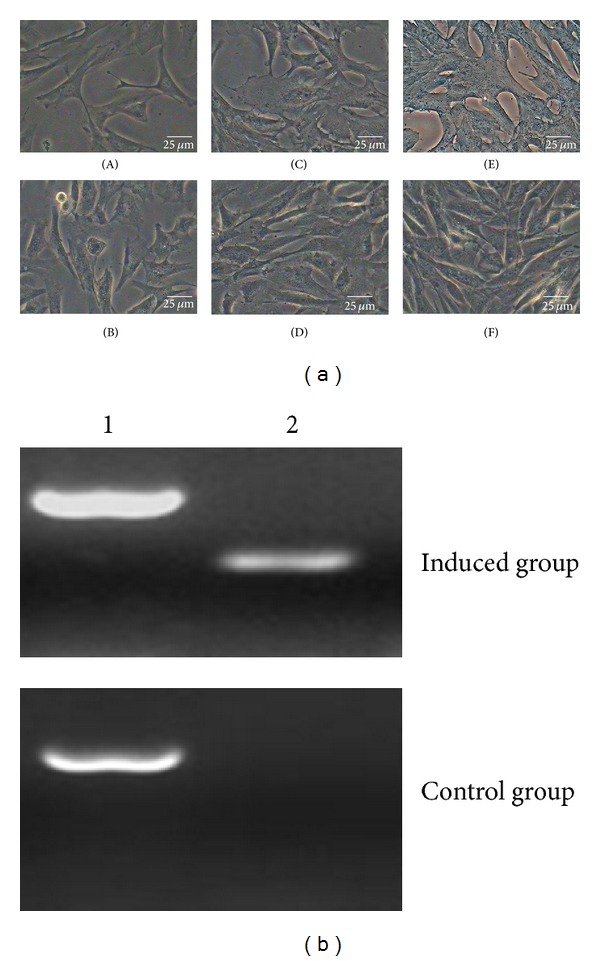
Cartilaginous differentiation of DMS/PCs. (a) DMS/PCs morphology and staining detection of the induced group and the control group. ((B), (D), and (F)) The control group of cartilaginous differentiation. Cells cultured in complete medium had no difference in the morphology and phenotype and were also negative for Alcian blue staining. (A) After 1 d, DMS/PCs in the induced group had no difference in the morphology and phenotype. (C) The induced group of cartilaginous differentiation. After induction for two weeks, the cells became compressed. (E) The cells of induced group were positive for Alcian blue staining (bar = 25 *μ*m). (b) RT-PCR detection of the cartilaginous markers COLII expression in induced group and control group. Induced cells were positive for COLII, but the control cells were not. 1: GAPDH; 2: COLII.

The ability of sheep DMS/PCs to differentiate into cartilaginous cells was detected, and the morphological and phenotypic analysis was carried on for the induced cells. The cell morphology changed at two weeks after induction; the cells became compressed (Figures 8(A)–8(C)) and were positive for Alcian blue staining (Figures 8(A)–8(E)). However, the cells which were cultured after 1 d, 7 d, and 14 d in the control group did not show the above effects (Figures 8(A), 8(B), 8(D), and 8(F)).

The result of cartilaginous differentiation of DMS/PCs was also analyzed by RT-PCR. Osteogenic-specific genes COLII were expressed in the induced group, but not in the control group (Figure 8(b)).

#### 3.6.6. Neurogenic Differentiation

The capacity for sheep DMS/PCs to differentiate into neurogenic cells was tested by induction with all-*trans*-retinoic acid and 2-mercaptoethanol; the morphological and phenotypic difference was observed for induced cells. After incubation in neural differentiation medium for 10 days, DMS/PCs exhibited neuritis from elongated shape, while there was no obvious morphological change in the control group.

**Figure 9:**
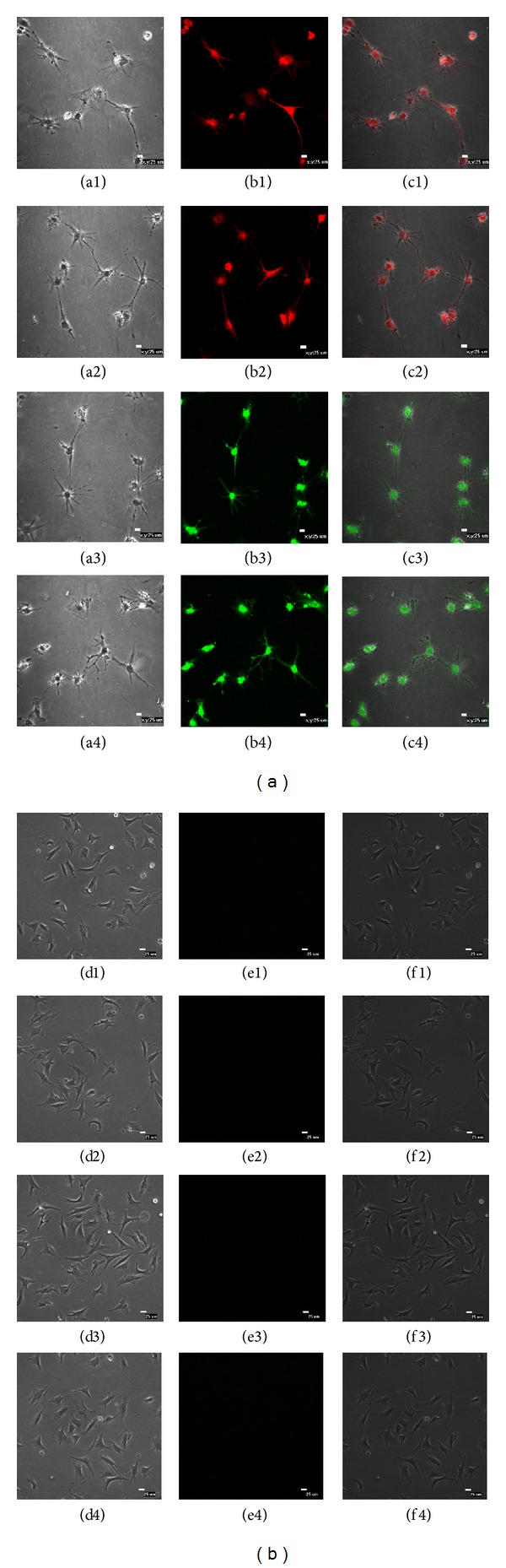
Neurogenic differentiation of DMS/PCs. After 10 days of induction, neural-like cells with a multipolar spindle-like shape were observed. Neural-specific markers including *β*-III tubulin (red), NF (red), nestin (green), and MAP-2 (green) were detected through immunofluorescence. (a) DMS/PCs morphology and identification of endothelial cells by immunofluorescent labeling. ((a1), (a2), (a3), and (a4)) Phase contrast. ((b1), (b2), (b3), and (b4)) *β*-III tubulin+, NF+, nestin+, and MAP-2+. ((c1), (c2), (c3), and (c4)) Merge (bar = 25 *μ*m). (b) The cells in the control group did not express the neurogenic-specific makers. ((d1), (d2), (d3), and (d4)) Phase contrast. ((e1), (e2), (e3), and (e4)) *β*-III tubulin-, NF−, nestin−, and MAP-2−. ((f1), (f2), (f3), and (f4)) Merge (bar = 25 *μ*m).

Furthermore, neurogenic-specific makers were detected using immunofluorescence. The neural cell markers *β*-III tubulin, NF, nestin, and MAP-2 were expressed positively by immunofluorescence (Figure 9(a)). However, the cells in the control group did not express the neurogenic-specific makers (Figure 9(b)). These results indicate that DMS/PCs can differentiate into neurocytes.

## 4. Discussion

In healthy individuals, skin integrity is maintained by stem cells in the skin which self-renew and generate daughter cells that undergo terminal differentiation. Despite accumulation of senescence markers in aged skin, various types of stem cells are maintained at normal levels throughout life. Skin ageing is induced by impaired stem cell mobilization or reduced number of stem cells is able to respond to proliferative signals. In the skin, existence of several distinct stem cell populations has been reported. Multipotent cells (skin-derived precursor cells) were present in human dermis; dermal stem cells represent 0.3% among human dermal foreskin fibroblasts. A resident pool of progenitor cells exists within the sebaceous gland, which is able to differentiate into both sebocytes and interfollicular epidermis. The self-renewal and multilineage differentiation of skin stem cells make these cells attractive not only for ageing process studies but also for regenerative medicine, tissue repair, gene therapy, and cell-based therapy with autologous adult stem cells not only in dermatology. In addition, they provide in vitro models to study epidermal lineage selection and its role in the ageing process [26].

In this study, DMS/PCs have successfully been isolated from the dorsal dermis of one-month-old sheep embryos using the way of trypsin digestion. All the results showed that the biological characteristics of the newly isolated stem cells were stable. Epidermal stem cell, dermal stem/progenitor cells, and some fibroblasts are present together, however, the cell type is thomogeneous through purification for 2-3 passages. By the reason of high activity of stem cells from the younger embryos, we choose one-month-old sheep embryos as our experimental materials. Therefore, DMS/PCs were cultured successfully in vitro and kept high activity for at least 48 passages. In addition, according to the regular program, the biological property of DMS/PCs containing growth dynamics, karyotype analysis, the detection of specific markers, and differentiation potentiality were performed on, respectively.

DMS/PCs growth dynamics were detected through growth curve drawing. Growth curve is a conventional method to detect cell growth rhythm, which has been widely used at home and abroad. The result of test showed that the growth curve had a typical “S” shape and had a normal population doubling time. Karyotype analysis is a major method for distinguishing normal cells from variants. The DMS/PCs cultured in this study were all normal diploids and the genetic property of the cells was stable during the passaging sequentially.

Mesenchymal stem cells preserve tissue-specific differences under in vitro culture conditions [27]. The markers identifying quiescent DMS/PCs include *β*-integrin, CD71, CD73, CD90, CD105 [28], and CD44 which resemble the specific makers of BMSCs. We examined the expression of the makers by RT-PCR and immunofluorescence. All the results showed that the DMS/PCs positively expressed *β*-integrin, CD71, CD44, and CD73. *β*-Integrin plays a fundamental role in cell adhesion and migration, linking the extracellular matrix to the actin cytoskeleton. In the adult, *β*-integrin is essential for a variety of biological processes including wound healing, leukocyte trafficking, and angiogenesis and is thus increasingly attractive therapeutic targets for a variety of conditions, notably cancer. CD71 is a member of the transferrin receptor family that is required for the import of iron into cells and is regulated in response to intracellular iron concentrations. Low iron concentrations increase the levels of transferrin receptors to increase iron intake into cells. Thus, the transferrin receptor maintains cellular iron homeostasis. CD44 plays a major role in many physiological processes, including cell substrate, interaction cell-cell adhesion, lymphocyte homing, and tumor metastasis. It has been reported that highly expressed CD44 in certain types of tumors is related to the hematogenic spread of tumor cells [29]. CD73, also known as ecto-5′-nucleotidase, is an enzyme used as a marker of lymphocyte differentiation [30].

Multilineage differentiation potentiality is the most notable characteristic of stem cells. In vitro, under the action of some inducing factors, the expressing of some key genes in the signaling pathway related to stem cell differentiation can change. Consequently, differentiation in specific directions was achieved. For instance, the WNT signaling pathway controls stem cell fate. WNT signals enlist two functionally and chemically different gene coactivators to direct the time and type of replicative divisions [31].

Dermal stem cells lie in the hair papillae, around pericytes, and elsewhere among other dermal cells. These form pericytes, myoblasts, fibroblasts, chondrocytes, and other specialized dermal cells. DMS/PCs may help to heal wounds, repair damaged tissues, regenerate aged skin, and reinvigorate growth of skin, hair, nails, and mucous membranes. In consideration of pluripotency of DMS/PCs, the induced differentiation experiment of DMS/PCs was performed on in vitro. Results showed that DMS/PCs were differentiated into adipocytes, osteoblasts, chondrocytes, and neurocytes and then examined relevant gene expression of these cell types. Some critical inducing factors have important functions for cell diferentiation, insulin, IBMX, and indomethacin were used to induce DMS/PCs to diferentiate into adipocytes; dexamethasone and *β*-glycerophosphate were used to induce DMS/PCs to differentiate into osteoblasts; TGF-*β*3 and bFGF were used to induce DMS/PCs to differentiate into chondrocytes; 2-mercaptoethanol and all-*trans*-retinoic acid were used to induce DMS/PCs to differentiate into neurocytes. And the roles of the inducing factors mainly affected the cell signaling pathways; some of the genes expressing that are overactive or underactive are changed. In addition, DMS/PCs can carry out the cross layers differentiation; DMS/PCs derived from mesoblastema can be induced to differentiate into mesodermal and ectodermal cells.

The above results suggested that sheep DMS/PCs not only had a strong self-renewal ability, but also had the potential to differentiate towards mesodermal and ectodermal cells. Therefore, DMS/PCs have become an ideal cell source in tissue engineering and clinical application.

## 5. Conclusions

In conclusion, an optimal method for isolation and culture of DMS/PCs was established in this study and cell morphology, surface markers, and biological characteristics were observed and detected. We also verified the multipotency of DMS/PCs and they could be induced to differentiate into adipocytes, osteoblasts, chondrocytes, and neurocytes. These results not only have provided a technological platform for the establishment of a sheep DMS/PCs line, but also propose a new method to preserve the valuable genetic resources of livestock and poultry.

## Figures and Tables

**Table 1:** Primer information for DMS/PCs identification.

Gene	Primer sequence	Tm (°C)	Cycle	Fragment size (bp)
GAPDH	F: 5′-GAAGGTCGGAGTGAACGGATT-3′	60.0	30	517
	R: 5′-GGTCATAAGTCCCTCCACGAT-3′			

*β*-Integrin	F: 5′-GTTTCACTTTGCTGGAGATGG-3′	58.0	30	243
	R: 5′-GCAGATAATGTTCCTACCGCT-3′			

CD71	F: 5′-GAGACTTCTTCCGTGCTACAT-3′	62.0	30	723
	R: 5′-GCTACCTTCCTCCTCCAACTC-3′			

CD44	F: 5′-GTGTCGTGTGCCCAGTTATGA-3′	60.0	30	511
	R: 5′-CTCGTCAGAGGTCCCATTTTC-3′			

CD73	F: 5′-GAGCAAGTGTGTCAACGCCAG-3′	61.9	30	569
	R: 5′-CATCCACGCCCTTCACTTTCT-3′			

PPAR-*γ*	F: 5′-ATCTTCCAGGGGTGTCAGTTT-3′	58.0	30	101
	R: 5′-TGGTCATTCAAGTCAAGGTTC-3′			

LPL	F: 5′-ACTATCCCCTGGGTAATGTGC-3′	60.0	30	283
	R: 5′-GGTTGGAAAGTGCCTCCGTTA-3′			

OPN	F: 5′-TTTCACTCCACCTTTCCCTAC-3′	58.5	30	489
	R: 5′-CCACCCTGCTTTAACATATCC-3′			

COLI	F: 5′-CCCAGTTGTCTTACGGCTATG-3′	60.0	30	323
	R: 5′-ACCTCTGTGTCCCTTCATTCC-3′			

ALP	F: 5′-CGAAGCACAAGCACTCTCACT-3′	60.0	30	419
	R: 5′-GGGAGCCAGACCAAAGATAGA-3′			

COLII	F: 5′-AAGTGGGGCGAGACTGTGATT-3′	60.0	30	281
	R: 5′-GGTTCTGGGTTCGGGTTTTTA-3′			
